# Naphthoquinone-Based Meroterpenoids from Marine-Derived *Streptomyces* sp. B9173

**DOI:** 10.3390/biom10081187

**Published:** 2020-08-15

**Authors:** Xinqian Shen, Xiaozheng Wang, Tingting Huang, Zixin Deng, Shuangjun Lin

**Affiliations:** State Key Laboratory of Microbial Metabolism, and Joint International Research Laboratory on Metabolic & Developmental Sciences, and School of Life Sciences & Biotechnology, Shanghai Jiao Tong University; 800 Dongchuan Rd, Shanghai 200240, China; asznpb@sjtu.edu.cn (X.S.); wangxiaozheng@sjtu.edu.cn (X.W.); zxdeng@sjtu.edu.cn (Z.D.)

**Keywords:** naphthoquinone, meroterpenoid, flaviogeranin family, natural products

## Abstract

Naphthoquinone-based meroterpenoids are hybrid polyketide-terpenoid natural products with chemical diversity and a broad range of biological activities. Here, we report the isolation of a group of naphthoquinone-containing compounds from *Streptomyces* sp. B9173, and their structures were elucidated by using a combination of spectroscopic techniques, including 1D, 2D NMR, and high-resolution mass (HRMS) analysis. Seven flaviogeranin congeners or intermediates, three of which were new, have been derived from common naphthoquinone backbone and subsequent oxidation, methylation, prenylation, and amino group incorporation. Both flaviogeranin B1 (**1**) and B (**2**) contain an amino group which was incorporated into the C8 of 1,3,6,8-terhydroxynaphthalene (THN). Flaviogeranin D (**3**) contains an intact *C*-geranylgeranyl residue attached to the C2 of THN, while the *O*-geranylgeranyl group of **2** links with the hydroxyl on the C2 site of THN. Four compounds were selected and tested for antibacterial activity and cytotoxicity, with **3** and flaviogeranin C2 (**5**) displaying potent activity against selected bacteria and cancer cell lines. In light of the structure features of isolated compounds and the biosynthetic genes, a biosynthetic pathway of naphthoquinone-based flaviogeranins has been proposed. These isolated compounds not only extend the structural diversity but also represent new insights into the biosynthesis of naphthoquinone-based meroterpenoids.

## 1. Introduction

Marine-derived microbial natural products are great sources for the discovery and development of pharmaceutical agents [1,2,3]. Marine Streptomyces continue to be the predominant producers, accounting for up to 69% of new secondary metabolites isolated from marine bacteria in 2018 [4]. Meanwhile, the genome of each Actinomycetes indicates genetic potential to produce 20–30 secondary metabolites. The rapidly accumulating microbial genomic information and developing bioinformatics platforms are further stimulating the revolution for natural product discovery [5]. However, the biosynthetic potential of microbial natural products is significantly underestimated based on the isolated compounds. It is hampered by the laboratorial cultivation methods, since many of these “cryptic” gene clusters are only expressed under certain culture conditions. Additionally, metabolite production is strongly influenced by growth conditions, including production media, pH, temperature, etc. [6,7].

**Figure 1:**
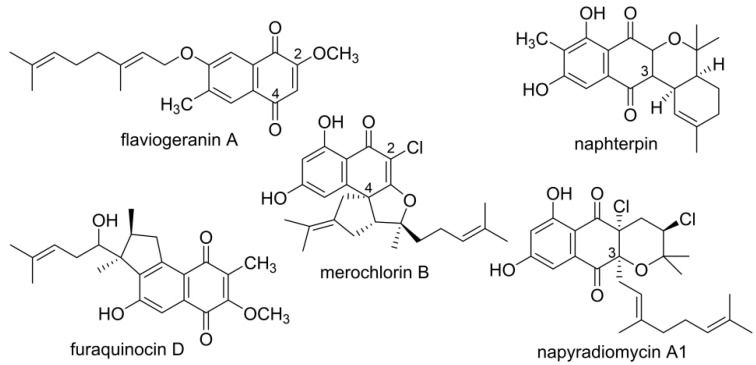
Representative naphthoquinone-based meroterpenoids.

Naphthoquinone-based terpenoids are natural products of mixed biosynthetic origin and exhibit antimicrobial, cytotoxic, antioxidant, and neuroprotective activity [8]. Examples include flaviogeranin, furaquinocin, naphterpin, naphthomevalin, and merochlorins, etc. [9] (Figure 1). Flaviogeranin is a neuroprotective agent against glutamate toxicity and is becoming a candidate for treatment of cerebral ischemic disorder [10]. Furaquinocins show cytocidal activity against melanoma cells in vitro [11]. These naphthoquinone-based meroterpenoids contain a 1,3,6,8-tetrahydroxynaphthalene (THN) core formed by a type III polyketide synthase (PKS) using malonyl-CoA as a substrate [12,13,14,15,16]. The prenyl moieties are suggested to play important roles in expanding structural diversity and enhancing biological activities. Simple prenyl groups are derived from geranyl diphosphate (GPP) or dimethylallyl diphosphate (DMAPP), and geranylgeranyl diphosphate gives rise to diterpenoids. Prenyltransferases catalyze the attachment of the corresponding polyprenyl group to the THN core [17]. The C3 of THN is not as nucleophilic as C2 and C4. Therefore, THN-derived meroterpenoids can be divided into C2/C4- prenylation (class I) and C3 prenylation pattern (class II). Successive steps such as methylation, hydroxylation, and chlorination are catalyzed by multiple modifying enzymes during the biosynthetic procedure, further enriching the structural diversity of the final products. 

**Figure 2:**
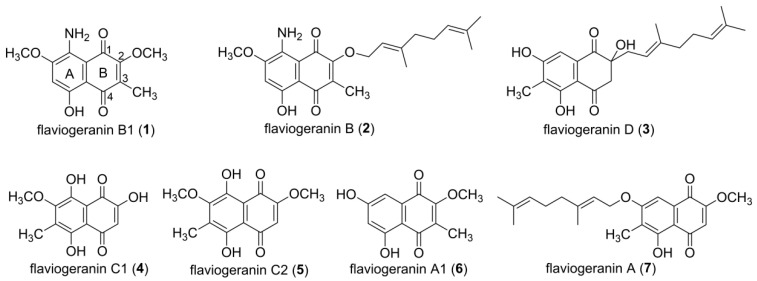
Structures of flaviogeranin congeners isolated from *Streptomyces* sp. **1–3** are new compounds and **4**–**7** are known compounds.

Our natural products project is devoted to the discovery of new bioactive metabolites from marine-derived actinomycetes. *Streptomyces* sp. B9173 was isolated from sea sediment collected from Chile. By optimization of growth conditions of *Streptomyces* sp. B9173, several indole alkaloids, such as maremycins, FR900452s, tryptanthrin, and meisoindigo, have been identified [18,19,20,21]. An in-depth bioinformatic investigation of secondary biosynthetic machineries of the whole genome yielded 31 gene clusters, clearly implying the biosynthetic potential of this strain [22]. Here, we report the isolation and structure determination of a group of flaviogeranin derivatives and pathway intermediates from *Streptomyces* sp. B9173 (Figure 2). Three previously undescribed compounds (**1**–**3**) with different oxidations, methylation, or prenylation on the THN core show unique characteristics compared with the known flaviogeranin. Among them, **1** and **2** are the only members containing an amino group at the C8 of THN instead of the hydroxyl group. These features expand the structural diversity of the flaviogeranin family of natural products, and these compounds provide evidence of the biosynthetic pathway. The new compound **3** showed potent antibacterial and cytotoxic activity.

## 2. Materials and Methods 

### 2.1. General Experimental Procedures

All ^1^H, ^13^C, and 2D NMR (HSQC, ^1^H-^1^H COSY, HMBC) spectral data were obtained in CDCl_3_ with a Bruker Avance 600 MHz spectrometer (Bruker, Rheinstetten, Germany). HPLC was performed on an Agilent 1260 Infinity LC (Agilent Technologies, Santa Clara, CA, USA) by using a Luna C18 5-µm column (4.6 × 250 mm) (Phenomenex, Torrance, CA, USA) at a flow rate of 0.5 mL/min, which was used for the routine analysis of extracts and fractions. High-resolution mass spectral data were obtained using a 6530 Q-TOF mass spectrometer coupled to an Agilent 1260 Infinity LC. Electrospray Ionization Mass Spectrometry (ESI-MS) was carried out on an Agilent 1100 LC/MS system with a Luna C18 5-µm column (4.6 × 150 mm, flow rate 0.5 mL/min). Sequence analyses and homologue searching were performed with PSI-BLAST search and anti-SMASH bacterial version. 

### 2.2. Bacterial Strains, Culture Conditions, and Biochemicals

Producer strain Streptomyces sp. B9173, isolated from a marine sediment sample collected at the Pacific coast of Chile, was kindly provided by Prof. Ralf Thiericke (Hans Knoell Institute, Jena, Germany). Streptomyces sp. B9173 was cultured in liquid tryptic soy broth (TSB) for mycelium or solid MS (2% soy flour, 2% mannitol, 1.5% agar, pH 7.2) medium for sporulation. For the production of flaviogeranin congeners, Streptomyces spores were inoculated into TSB medium in an Erlenmeyer flask as seed culture and incubated at 28 °C with shaking at 200 rpm. Then, fermentation medium was inoculated with 8% (*v/v*) 2-day seed culture for a continuing 7 day. Production of the secondary metabolites was monitored at various time points during fermentation and analyzed by HPLC-MS. 

HPLC-grade solvents were used for all chromatographic analyses. For column chromatography (CC), Sephadex LH-20 (GE Healthcare Bio-Science AB, Pittsburgh, PA, USA), silica gel (200–300 mesh, 300–400 mesh, Tsingtao Marine Chemical Co. Ltd., Tsingtao, China), and RP-C18 (ODS-A, 50 µm, YMC, Kyoto, Japan) were used. Other common biochemicals were purchased from standard commercial sources. 

### 2.3. Extraction and Isolation of Flaviogeranin Congeners

For small-scale analyses, the Streptomyces cultures were clarified by centrifugation after fermentation. The metabolites were extracted three times with ethyl acetate (EtOAc) from supernatant, concentrated by rotary evaporation, and the resulting residue was dissolved in methanol prior to HPLC-MS analysis, with 0.1% formic acid as solvent A and acetonitrile (ACN) as solvent B. A 46-min gradient elution condition for HPLC analysis started with a linear gradient from 10% B to 60% B for 13 min, followed by a gradient from 60–95% B for 22 min, and an additional isocratic step for 5 min. The column was washed by increasing the gradient to 100% B and was equilibrated for 5 min, followed by equilibration for 5 min under the starting conditions (10% B).

Large-scale fermentation (150 L) was performed using 2-L baffled flasks containing 0.5 L liquid MS medium with 8% seed culture. The cell pellet was separated from fermentation broth by centrifugation. The supernatant was extracted three times with half volume of EtOAc, combined together and evaporated under reduced pressure to yield a 27.1 g dark brown crude extract. The obtained EtOAc crude extract was loaded onto a silica gel column and eluted using an increasing gradient of 0% to 100% petroleum ether-CH_2_Cl_2_ and CH_2_Cl_2_-MeOH as mobile phase. Every 200 mL was collected as a fraction at the 10 mL/min flow rate. In total, 12 fractions (Fr.1–Fr.12) were collected and analyzed by HPLC. Fr.3 (2.1 g) was further purified using flash RP-C18 column chromatography with an increasing gradient of 20% to 50% MeOH to give 15 fractions (Fr.3.1–Fr.3.15). Fr3.9 (118.2 mg) was purified by a Sephadex LH-20 column packed with Sephadex LH-20 resin (40 g) and eluted with MeOH and acetone, respectively, to yield **6** (13.7 mg) and **7** (18.3 mg). Fr.3.10 (72.6 mg) was separated by a Sephadex LH-20 column and eluted with MeOH to yield **3** (15.2 mg). Fr.3.8 (853.1 mg) was subjected to an RP-C18 column, eluted with 50%MeOH in H_2_O and followed by Sephadex LH-20 separation to yield **1** (11.1 mg), **2** (10.8 mg), **4** (17.7 mg), and **5** (15.7 mg). The NMR data for new compounds (**1**–**3**) are presented in Figures S3–S5 and summarized in Table 1, Table 2 and Table 3. The ^1^H-NMR spectra and HR-ESI MS data of known compounds (**4**–**7**) are listed in Figures S6–S9 and Table S1, which are matched by the literature [10,23,24,25,26].

### 2.4. Bioactivity Assay

The antibacterial activities were tested against *Streptococcus aureus* ATCC 43,300 and *Mycobacterium smegmatis* MC^2^ 255 using a gradient dilution assay using 96-well plates. The compounds were dissolved in DMSO to 10 mg/mL as a stock solution and diluted in culture media to 1 μg/mL as the initial concentration. The 96-well plates with bacterial inoculation were incubated at 37 °C for 8–12 h. Microbial growth was measured by monitoring OD_600_ on a Synergy 2 multi-mode microplate reader (BioTek, Winooski, VT, USA). Erythromycin was used as a positive control. 

The compounds were tested for cytotoxic effects with A549 (non-small-cell lung carcinoma cells) and HeLa cell lines by 3-[4,5-dimethylthiazole-2-yl]-2,5-diphenyltetrazolium bromide (MTT) assay with minor modifications. The A549 cell line was grown in F-12K media with 10% fetal bovine serum at 37 °C under a humidified atmosphere of 5% CO_2_. Subsequently, 10 uL diluted solutions of compounds were added to 90 μL aliquots of cell suspension (5 × 10^4^ cells/mL) in 96-well plates after overnight incubation, and incubation was continued for 72 h. The MTT solution (10 μL 5 mg/mL) was pipetted into each well and incubated for an additional 4 h. The medium containing MTT was gently removed and DMSO (150 μL) was added in order to dissolve formed formazan crystals. OD_490_ was detected by a microplate reader and the data were analyzed by GraphPad Prism 5.0. Doxorubicin was used as a positive control. Data are presented as average values from at least duplicate experiments.

## 3. Results

### 3.1. Structural Elucidation of Naphthoquinone Family Metabolites

Meroterpenoids are hybrid natural products harboring structurally various terpenoid and non-terpenoid moieties which diversify the chemical structures and biological activities. Flaviogeranin is a prenylated naphthoquinone derivative featuring a polyketide-derived naphthoquinone unit and a geranyl side chain [10]. Generally, the secondary metabolite profile of the certain strain is medium-dependent. We had previously investigated marine-derived *Streptomyces* sp. B9173 and found that it produced a variety of metabolites under different culture conditions. The growth and sporulation of *Streptomyces* sp. B9173 occurs extremely well on MS medium plate, encouraging us to isolate new natural products in this medium (Figure S1). The strain was cultivated in MS medium at 28 °C for 7 days, extracted with a half volume of EtOAC three times, and subsequent column chromatography resulted in the isolation of seven compounds (Figure 2). Among these naphthoquinone-containing compounds, three were new, with various modifications including methylations and prenylations or an unusual amination on the benzene moiety (ring A). Each of the new compounds is named “flaviogeranin” after the prenylated THN scaffold. 

**Figure 3:**
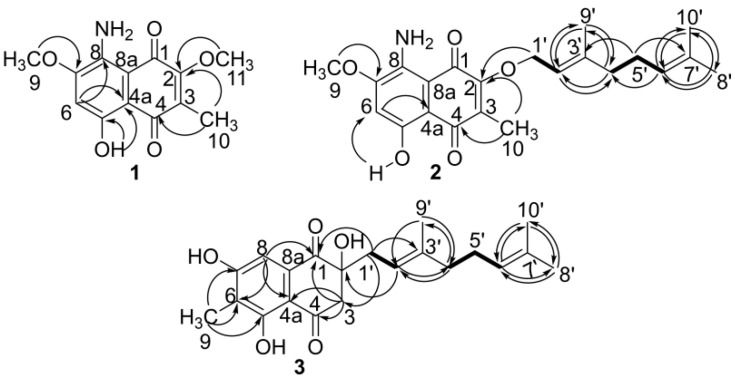
^1^H-^1^H COSY (bold lines) and HMBC correlation (arrows) supporting planar structures of **1**–**3**.

Flaviogeranin B1 (**1**) was obtained as a violet powder. UV-vis (CH_3_Cl) *λ*_max_ (log *ε*): 242 (3.71), 304 (3.38), 504 (3.34), 536 (3.54), 576 (3.5) nm; high-resolution electrospray ionization mass (HRESIMS) analysis for **1** afforded an [M + H]^+^ ion at *m/z* 264.0868, and the molecular formula of **1** can be deduced as C_13_H_13_NO_5_ (calcd. 264.0866 for [M + H]^+^). Interpretation of the ^1^H, ^13^C, DEPT, and HSQC NMR spectra of **1** in CDCl_3_ revealed one aromatic methine signal (*δ_C_* 104.3, C-6 and *δ_H_*, 6.48, H-6), two methoxy groups (*δ_C_* 56.4, C-9 and *δ_H_* 3.97, H-9; *δ_C_* 61.0, C-11, and *δ_H_* 4.07, H-11), and one methyl group (*δ_C_* 8.83, C-10 and *δ_H_*, 2.09, H-10). Further analysis of the 1D NMR data revealed the existence of one hydroxyl group at *δ_H_* 14.41 that should form a hydrogen bond with a carbonyl group, two carbonyl carbons (*δ_C_* 186.8, C-4; *δ_C_* 180.1, C-1), and seven nonprotonated double-bond carbons (*δ_C_* 159.4, C-2; *δ_C_* 131.6, C-3; *δ_C_* 106.1, C-4a; *δ_C_* 160.3, C-5; *δ_C_* 155.4, C-7; *δ_C_* 139.9, C-8; *δ_C_* 107.5, C-8a). Thus, **1** was identified as a naphthoquinone bearing a hydroxy group, an amino group, one methyl group, and two methoxy groups. The analysis of the HMBC spectrum revealed the correlations from H-6 to C-4a and C-8; OCH_3_-9 to C-7; CH_3_-10 to C-2 and C-4; OCH_3_-11 to C-2. The positions of other substituent groups have been established by NMR, expect the NH_2_ at C8. According to the furaquinocin biosynthesis, 8-amino flavin has been confirmed as a cryptic intermediate [23]. Therefore, the amino group is speculated to attach at the C8. Finally, the structure of **1** was established as 8-amino-5-hydroxy-2,7-dimethoxy-3-methylnaphthalene-1,4-dione (Figure 3 and Figure S3 and Table 1).

Flaviogeranin B (**2**) was obtained as a violet powder. UV-vis (CH_3_Cl) *λ*_max_ (log *ε*): 242 (3.40), 304 (3.07), 504 (3.06), 536(3.27), 576(3.23) nm; HRESIMS analysis afforded an [M + H]^+^ ion at *m/z* 386.1969, giving the molecular formula of **2** as C_22_H_27_NO_5_ (calcd. 386.1962 for [M + H]^+^). The 1D NMR data of **2** were similar to those of **1**, but there were ten more carbon signals including four double-bond carbons (*δ_C_*: 119.8 (d); 142.6 (s); 123.7 (d); 131.8 (s)), three CH_2_ (*δ_C_*: 69.8 (t); 39.6 (t); 26.3 (t)), and three CH_3_ (*δ_C_*: 25.6 (q); 16.5 (q); 17.6 (q)), indicating that **2** contains a geranyl group. A dimethylallyl group in **2** was constructed by HMBC from two allylic methyls (*δ_H_* 1.65, H-8′; *δ_H_* 1.58, H-10′) to *δ_C_* 131.8, C-7′ and *δ_C_* 123.7, C-6′ and a COSY cross-peak between *δ_H_* 5.05, H-6′ and *δ_H_* 2.03, H-5′. Another isoprene unit was identified by HMBCs from *δ_H_* 1.70 (s, CH_3_, H-9′) and *δ_H_* 2.06 (m, CH_2_, H-4′) to *δ_C_* 142.6 (C-3′) and *δ_C_* 119.8 (C-2′) and a vicinal coupling between *δ_H_* 5.49 (m, CH, H-2′) and *δ_H_* 4.87 (d, 2H, H-1′). The two units were joined between *δ_H_* 2.06 (H-4′) and *δ_H_* 2.03 (H-5′) through COSY spectrum analysis. A geranyl group thus obtained was connected to 2-*O* from the correlation between *δ_H_* 4.87 (H-1′) and *δ_C_* 158.7 (C-2). Thus, **2** was established as (*E*)-8-amino-3-((3,7-dimethylocta-2,6-dien-1-yl)oxy)-8-hydroxy-6-methoxy-2-methylnaphthalene-1,4-dione (Figure 3, Figure S4 and Table 2).

Flaviogeranin D (**3**) was obtained as orange oil. [*α*]^23^_D_ +16.3 (c 0.10, CH_3_Cl); UV (CH_3_Cl) *λ*_max_ (log *ε*): 256 (3.90), 310 (3.46), 353 (3.34) nm; HRESIMS analysis afforded an [M + H]^+^ ion at *m/z* 359.1839, giving the molecular formula of **3** as C_21_H_26_O_5_ (calcd. 359.1824 for [M + H]^+^). The ^13^C and DEPT NMR spectra of **3** suggested the presence of four methyl (*δ_C_* 8.1, C-9; *δ_C_* 16.1, C-9′; *δ_C_* 25.6, C-8′; *δ_C_* 17.6, C-10′), one oxygenated quaternary carbon (*δ_C_* 77.9, C-2), four CH_2_ (*δ_C_* 49.1, C-3; *δ_C_* 38.9, C-1′; *δ_C_* 39.8, C-4′; *δ_C_* 26.3, C-5′), two carbonyls (*δ_C_* 199.7, C-1; *δ_C_* 198.9, C-4), three olefin CH (*δ_C_* 116.0, C-2′; *δ_C_* 123.9, C-6′; *δ_C_* 106.5, C-8), and seven nonprotonated double-bond carbons (*δ_C_* 112.0, C-4a; *δ_C_* 161.2, C-5; *δ_C_* 119.2, C-6; *δ_C_* 162.1, C-7; *δ_C_* 131.4, C-8a; *δ_C_* 141.0, C-3′; *δ_C_* 123.9, C-6′). A reduced naphthalene-1,4-dione substructure was constructed through analysis of the HMBC correlation from *δ_H_* 7.07 (H-8) to *δ_C_* 119.2 (C-6), *δ_C_* 112.0 (C-4a) and *δ_C_* 199.7 (C-1); *δ_H_* 2.19 (H-9) to *δ_C_* 119.2 (C-6), *δ_C_* 162.1 (C-7) and *δ_C_* 161.2 (C-5); *δ_H_* 3.23 and 3.13 (d, *J* = 16.8 Hz, H-3) to *δ_C_* 198.9 (C-4), *δ_C_* 112.0 (C-4a), and *δ_C_* 199.7 (C-1). A geranyl group was identified in comparison with the NMR data of **2** and connected to C-2 (*δ_C_* 77.9) based on the correlation from *δ_H_* 4.98 (C-2′) to *δ_C_* 77.9 (C-2); *δ_H_* 2.49, 2.38 (H-1′) to *δ_C_* 199.7 (C-1), *δ_C_* 77.9 (C-2), and 39.1 (C-3). Therefore, the structure of **3** was identified as (*E*)-2-(3,7-dimethylocta-2,6-dien-1-yl)-2,5,7-trihydroxy-6-methyl-2,3-dihydronaphthalene-1,4-dione (Figure 3, Figures S5, and S10 and Table 3).

### 3.2. Evaluation of Bioactivities 

In order to evaluate the structure–activity relationship, four compounds (**1**–**3**, and **5**) were selected and tested for their antibacterial activity and cytotoxicity (Table 4). The antibacterial activity was investigated by using a standard gradient dilution assay against strains *Staphylococcus aureus* and *Mycobacterium smegmatis*, using erythromycin as the positive control. As shown in Table 4, both **3** and **5** showed potent inhibitory activity against *S. aureus* and *M. smegmatis*, with the minimum inhibitory concentration (MIC) values ranging from 5 to 9 µg/mL, which were similar to the MIC of the positive control, erythromycin. However, the compounds harboring the amino group on the C8 (**1** and **2**) showed relatively weaker activity (MIC range from 12 to 35 µg/mL). 

Two cancer cell lines, HeLa and A549, were chosen to test the cytotoxicity, using doxorubicin as the positive control. Again, **3** and **5** exhibited potent cytotoxic activity towards both cell lines (IC_50_ ranging from 0.4 to 1.1 µM), while **1** and **2** were inactive (IC_50_ ranging from 25 to 50 µM). Taken together, **3** and **5** exhibited potent cytotoxicity and antibacterial activity. 

### 3.3. Isolated New Natural Products Revealing New Insight into Flaviogeranin Biosynthesis

Even though the molecular architecture of these isolated naphthoquinone-containing compounds is different, the common structural feature indicates that they may use the same early biosynthetic intermediates and biosynthetic pathway. Formation of the aromatic backbone of naphthoquinone utilizes a common precursor which is synthesized by type III PKS [12,13]. To mine the genome for the biosynthetic genes of these flaviogeranin congeners, we aimed at identifying type III PKS genes that are essential for the biosynthesis of the THN skeleton and genes encoding prenyltransferases in the adjacent region [22]. A ~48 kb candidate gene cluster has been found and sequence analysis revealed type III PKS genes putatively involved in the construction of the naphthoquinone polyketide core, associated with the biosynthesis and connection of the terpenoid units [22]. Putative regulators, resistance genes, and genes with unknown function are involved in the cluster too. 

THN formed by such type III PKS appears to be an intermediate in the biosynthetic pathways for various naphthoquinone-containing secondary metabolites [27]. Type III PKS from *Streptomyces* sp. B9173 showed 91% identity in amino acid sequence to the chalcone synthase (CHS)-like protein RppA from *Streptomyces griseus* [12]. Sequence alignment of the type III PKS with its counterparts from the gene cluster of naphthoquinone-containing compounds revealed the conserved catalytic triad residues (Cys138, His270, and Asn303) (Figure S2). The terpenoid origin of the C_10_ moiety indicated that the mevalonic acid (MVA) pathway genes in the cluster were responsible for the formation of isopentenyl pyrophosphate and dimethylallyl pyrophosphate building blocks. 

**Figure 4:**
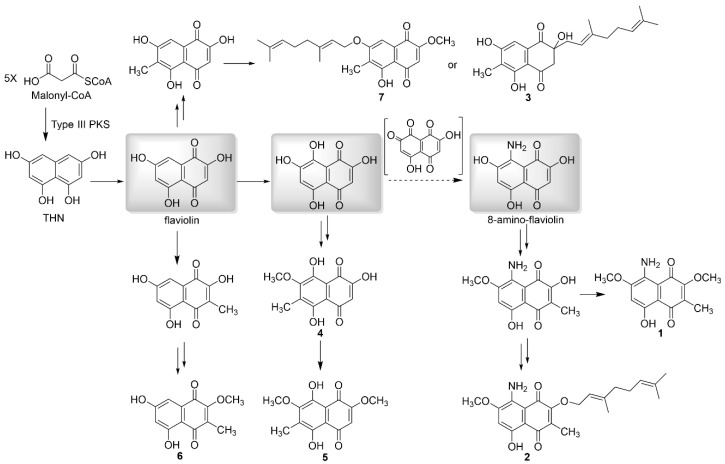
Proposed biosynthetic pathway of flaviogeranins supported by the isolation of isolated flaviogeranin intermediates and congeners. Three common intermediates of the divergent biosynthetic pathway are highlighted in gray.

Based on the chemical structures of these flaviogeranin congeners isolated from *Streptomyces* sp. B9173, we proposed a biosynthetic pathway, as shown in Figure 4. Type III PKS catalyzes naphthoquinone ring formation: it selects malonyl-coenzyme A as the start unit, carries out four successive extensions to form the pentaketide, releases the resulting nonreducing ketide, and cyclizes to form THN. The symmetric intermediate THN was subsequently oxidized to form 2,5,7-trihydroxy-1,4-naphthoquinone (flaviolin), which then modified to form various compounds through oxidation, methylation, and prenylations to produce **3**–**7**. Two putative prenyltrasnferases likely catalyze the prenylation at two different positions: the prenylation at hydroxyl group on the C7 to generate flaviogeranin A (**7**) and the prenylation at the C2 to give flaviogeranin D (**3**). Isolation of flaviogeranin C1 (**4**) and flaviogeranin C2 (**5**) revealed the oxidation at the C8, followed by successive methylations. An aminotransferase introduced an amino group on C8 to generate 8-amino-flaviolin (8-amino-2,5,7-trihydroxynaphthalene-1,4-dione) and dedicated modification enzymes further tailored the scaffold to form flaviogeranin B1 (**1**) and flaviogeranin B (**2**). The divergent pathway represents the formation of diverse architecturally flaviogeranin family compounds via the common THN precursor.

## 4. Discussion

Marine microbial metabolites are a prevalent source of bioactive natural products and a number of entities have been identified with promising therapeutic potential [1,28]. Taking natural naphthoquinone meroterpenoids, for example, much research has been carried out to test differences in their biological activity and elucidate their pharmacological profiles. In this work, we checked the metabolite profiles of marine-derived *Streptomyces* sp. B9173 in MS medium, set up the large-scale fermentation, and performed subsequent isolation. Chemical investigations into the fermentation cultures led to the isolation of a group of naphthoquinone-containing compounds. Among them, **1**–**3** are previously unreported naphthoquinone-containing derivatives, and others are biogenetically related compounds including flaviogeranin A and substituted 5-hydroxy-1,4-naphthoquinones [10,25,26]. 

The carbon skeleton of naphthoquinone has been established from several metabolic pathways. One is the direct incorporation of chorismate, derived from the shikimate pathway via formation of 1,4-dihydroxy-2-naphthoate intermediate [29] or through a futalosine intermediate [30]. The other pathway is through a common THN intermediate which was formed by type III PKS catalyzing Claisen and aldol condensation [12]. From the point view of the fundamental biosynthetic basis, naphthoquinone-derived meroterpenoids share a common paradigm in which the THN is formed and subsequently attached with a terpenoid moiety. THN is preceded by the biosynthesis of several hybrid meroterpenoids (furaquinocin and napyradiomycin), and previous precursor incorporation studies demonstrated the polyketide biosynthetic origin of THN. Additionally, it utilizes the mevalonate pathway for the production of IPP for terpenoids [31]. As seen in flaviogeranin biosynthesis, core enzymes including type III PKS and prenyltransferases contribute to the naphthoquinone-terpenoid skeleton formation while tailoring enzymes such as methyltransferases, oxidases, etc., accounting for the structural diversity. It is conceivable that hydroxylation at the C8 results in intermediate flaviogeranin C1 (**4**) and flaviogeranin C2 (**5**). Isolation of **1** and **2** suggests that 8-amino-flaviolin serves as a common precursor and an aminotransferase catalyzes the introduction of the amino group to the C8 of naphthoquinone core. A similar aminotransferase was seen in the furaquinocin biosynthetic pathway and 8-amino-flaviolin can be detected in the heterologous expression system consisting of three contiguous genes (Type III PKS, monooxygenase, and aminotransferase) [15,23]. Since no furaquinocin congeners with the C8 amino group have been reported until now, the isolation of the amino group containing compounds **1** and **2** in our work confirmed the “cryptic transamination” and indicated an independent biosynthetic pathway through the precursor 8-amino-flaviolin. 

The common and differentiating features of these flaviogeranin compounds are related to their bioactivities. The biological activities of four flaviogeranins (**1**–**3** and **5**) were tested for antibacterial activity and cytotoxicity. Both **3** and **5** show potent activity while **1** and **2**, with the amino group at the C8, exhibit weaker activity. The true function of transamination at the C8 of naphthoquinone remains unclear. It is suggested that such modification may confer self-protection for the producers or facilitate the catalytic efficiency of specific enzymes during biosynthesis. 

The remarkable structural variations observed among flaviogeranin congeners derive from the catalytic activities of diverse enzymes for structural decoration. Multiple modification enzymes function on the common intermediate THN to generate diverse flaviogeranin congeners. The isolated flaviogeranin congeners and intermediates further unveil the biosynthetic steps and new modification enzymes for flaviogeranin family synthesis. Meanwhile, the identified new flaviogeranin congeners with antibacterial activity and cytotoxicity may start to approach the development of lead compounds.

## 5. Conclusions

In summary, we have achieved the isolation of seven flaviogeranin congeners, including three new compounds, from marine-derived *Streptomyces* sp. B9173, based on the modified fermentation condition and the chemical profiling. Their structures were elucidated by using spectroscopic technologies, and two compounds were found to contain an amino group on the THN moiety, which is very rare in the flaviogeranin family. Identification of these two amino group-containing compounds (**1** and **2**) confirmed the cryptic transamination in the flaviogeranin biosynthetic pathway and provided the opportunity to investigate the incorporation of the amino group into the naphthoquinone ring. Antibacterial and cytotoxicity assays showed that **3** and **5** potently inhibit selected bacteria and cancer lines, respectively. The data also show a correlation between bioactivity and chemical structure. These isolated compounds broaden the understanding of the biosynthetic pathway and enzymatic reaction. 

## Figures and Tables

**Table 1:** ^1^H and ^13^C-NMR, ^1^H-^1^H COSY, gHMBC data of **1** (600 MHz, CDCl_3_).

No	δ_H_(Multi, *J*/Hz)	δ_C_(DEPT)	^1^H-^1^H COSY	gHMBC
1		180.1 (s)		
2		159.4 (s)		
3		131.6 (s)		
4		186.8 (s)		
4a		106.1 (s)		
5		160.3 (s)		
6	6.48 (1H,s)	104.3 (d)		5,7,4a,8
7		155.4 (s)		
8		139.9 (s)		
8a		107.5 (s)		
9	3.97 (3H,s)	56.4 (q)		7
10	2.09 (3H,s)	8.8 (q)		6,2
11	4.07 (3H,s)	61.0 (q)		2
5-OH	14.41 (brs)			4,4a

**Table 2:** ^1^H and ^13^C-NMR, ^1^H-^1^H COSY, gHMBC data of **2** (600 MHz, CDCl_3_).

No	δ_H_(Multi, *J*/Hz)	δ_C_(DEPT)	^1^H-^1^H COSY	gHMBC
1		180.4 (s)		
2		158.7 (s)		
3		132.5 (s)		
4		186.8 (s)		
4a		106.1 (s)		
5		160.1 (s)		
6	6.47 (1H,s)	104.3 (d)		5,7,4a
7		155.4 (s)		
8		139.7 (s)		
8a		107.5 (s)		
9	3.96 (3H,s)	56.4 (q)		7
10	2.09 (3H,s)	9.1 (q)		2,4
1′	4.87 (2H,d,7.2)	69.8 (t)	2′	2,3′
2′	5.49 (1H,m)	119.8 (d)	1′	9′,4′
3′		142.6 (s)		
4′	2.06 (2H,m)	39.6 (t)	5′	3′,2′,9′
5′	2.07 (2H,m)	26.3 (t)	4′,6′	3′,7′
6′	5.05 (1H,m)	123.7 (d)	5′	8′,4′,10′
7′		131.8 (s)		
8′	1.65 (3H,s)	25.6 (q)		7′,10′,6′
9′	1.70 (3H,s)	16.5 (q)		4′,2′,3′
10′	1.58 (3H,s)	17.6 (q)		7′,8′,6′
5-OH	14.42 (brs)			4a,5

**Table 3:** ^1^H and ^13^C-NMR, ^1^H-^1^H COSY, gHMBC data of **3** (600 MHz, CDCl_3_).

No	δ_H_(Multi, *J*/Hz)	δ_C_(DEPT)	^1^H-^1^H COSY	gHMBC
1		199.7 (s)		
2		77.9 (s)		
3	3.23 (1H,d,16.8) 3.13 (1H,d,16.8)	49.1 (t)		1,4a
4		198.9 (s)		
4a		112.0 (s)		
5		161.2 (s)		
6		119.2 (s)		
7		162.1 (s)		
8	7.07 (1H,s)	106.5 (d)		4a,6
8a		131.4 (s)		
9	2.19 (3H,s)	8.1 (q)		5,7
1′	2.49 (1H,dd,14.5,8.3) 2.38 (1H,dd,14.5,8.3)	38.9 (t)	2′	2,3′,1
2′	4.98 (1H,t,6.9)	116.0 (d)	1′	9′,4′
3′		141.0 (s)		
4′	1.94 (2H,m)	39.8 (t)	5′	3′,2′,9′
5′	2.02 (2H,m)	26.3 (t)	4′,6′	3′,7′
6′	5.03 (1H,t,7.5)	123.9 (d)	5′	8′,4′,10′
7′		131.3 (s)		
8′	1.67 (3H,s)	25.6 (q)		7′,10′,6′
9′	1.43 (3H,s)	16.1 (q)		4′,2′,3′
10′	1.57 (3H,s)	17.6 (q)		7′,8′,6′
4-OH	12.55 (brs)			3,4a,5

**Table 4:** The MIC values and IC_50_ values of 4 compounds against microorganism and tumor cell lines, respectively.

Compound	MIC (μg/mL)		IC_50_ (μM)	
	*S. aureus*	*M. smegmatis*	A549	Hela
1	14.6 ± 0.3	12.4 ± 0.3	25.7 ± 0.4	34.7 ± 0.6
2	28.1 ± 0.4	35.1 ± 0.6	46.6 ± 0.4	50.2 ± 0.5
3	9.2 ± 0.2	5.2 ± 0.2	0.6 ± 0.2	0.4 ± 0.2
5	8.1 ± 0.2	7.7 ± 0.2	0.9 ± 0.2	1.1 ± 0.3
erythromycin	7.6 ± 0.2	4.5 ± 0.2	NA	NA
doxorubicin	NA	NA	0.1 ± 0.0	0.5 ± 0.1

## Supplementary Materials

The following are available online at https://www.mdpi.com/2218-273X/10/8/1187/s1, Figure S1: HPLC profile of *Streptomyces* sp. B9173 metabolites in MS media, Figure S2: Sequence alignment of the type III PKS from gene clusters of naphthoquinone-containing compounds, Figure S3: NMR spectra of flaviogeranin B1 (**1**)**,** Figure S4: NMR spectra of flaviogeranin B (**2**), Figure S5: NMR spectra of flaviogeranin D (**3**), Figure S6: ^1^H NMR spectrum of flaviogeranin C1 (**4**), Figure S7. ^1^H NMR spectrum of flaviogeranin C2 (**5**), Figure S8. ^1^H NMR spectrum of flaviogeranin A1 (**6**), Figure S9. ^1^H NMR spectrum of flaviogeranin A (**7**); Figure S10. CD spectrum of flaviogeranin D; Table S1. HR-ESI MS of known compounds **4**-**7**.
