# Supplementary material for: Naphthoquinone-Based Meroterpenoids from Marine-Derived Streptomyces sp. B9173
Source: Biomolecules. 2020 Aug 15;10(8):1187. doi: 10.3390/biom10081187 (PMC7463872; doi:10.3390/biom10081187)
Supplement: Supplementary file 1 [file biomolecules-10-01187-s001.pdf]

Naphthoquinone-Based Meroterpenoids from Marine-Derived *Streptomyces* sp. B9173

Xinqian Shen †, Xiaozheng Wang †, Tingting Huang *, Zixin Deng and Shuangjun Lin *

State Key Laboratory of Microbial Metabolism, and Joint International Research Laboratory on Metabolic & Developmental Sciences, and School of Life Sciences & Biotechnology, Shanghai Jiao Tong University; 800 Dongchuan Rd, Shanghai 200240, China; asznpb@sjtu.edu.cn (X.S.); wangxiaozheng@sjtu.edu.cn (X.W.); zxdeng@sjtu.edu.cn (Z.D.)

* Correspondence: tingwing82@sjtu.edu.cn (T.H.); linsj@sjtu.edu.cn (S.L.); Tel.: +86-21-3420-4710 (S.L.)

† These two authors contribute equally to this work.

Supplementary information

Figure S1. HPLC profile of *Stretomyces* sp. B9173 metabolites in MS media.

Figure S2. Sequence alignment of the type III PKS from gene clusters of naphthoquinone containing compounds.

Figure S3. NMR spectra of faviogeranin B1 (**1**).

Figure S4. NMR spectra of flaviogeranin B2 (**2**).

Figure S5. NMR spectra of flaviogeranin D (**3**).

Figure S6. ^{1}H NMR spectrum of flaviogeranin C1 (**4**).

Figure S7. ^{1}H NMR spectrum of flaviogeranin C2 (**5**).

Figure S8. ^{1}H NMR spectrum of flaviogeranin A1 (**6**).

Figure S9. ^{1}H NMR spectrum of flaviogeranin A (**7**).

Figure S10. CD spectrum of flaviogeranin D (**3**).

Table S1. HR-ESI MS of known compounds **4-7**.

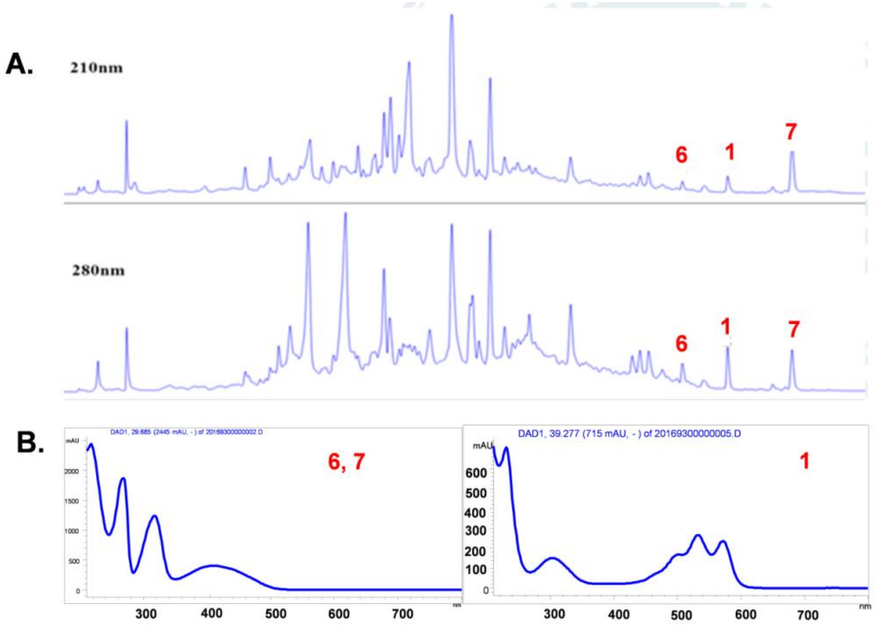

Figure S1. HPLC profile of *Stretomyces* sp. B9173 metabolites in MS media.

A. HPLC profile of *Stretomyces* sp. B9173 metabolites in MS media.

B. Specific UV spectrum of several naphthoquinone containing compounds.

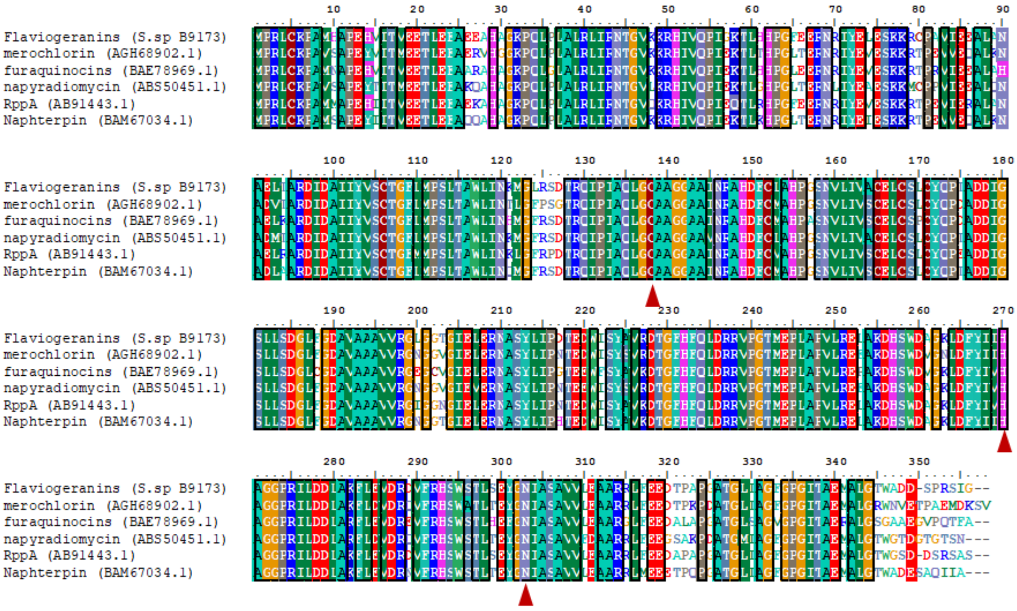

Figure S2. Sequence alignment of the type III PKS from gene clusters of naphthoquinone containing compounds.

Sequence alignment revealed the conserved catalytic triad residues (Cys138, His270, and Asn303). AGH68902.1: type-III polyketide synthase [*Streptomyces* sp. CNH189]; BAE78969.1: Fur1 [*Streptomyces*. sp KO-3988]; ABS50451.1: NapB1 [*Streptomyces aculeolatus*]; BAB91443.1:Type III polyketide synthase RppA [*Streptomyces antibioticus*]; BAM67034.1: 1,3,6,8-tetrahydroxynaphthalene synthase [*Streptomyces* sp. CL190].

A.

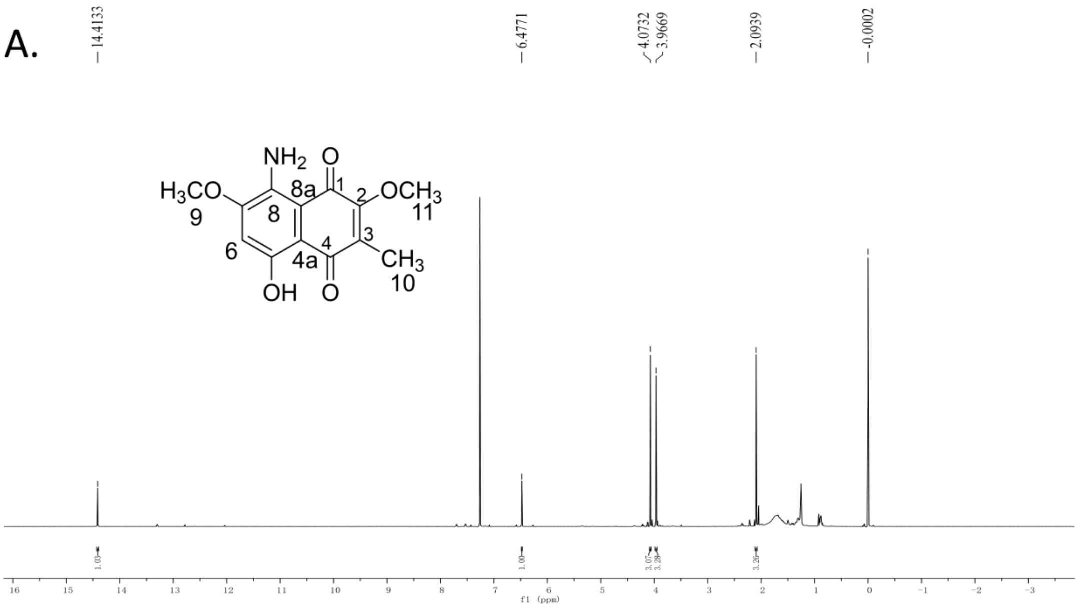
14.4133
6.4771
4.0732
3.9669
2.0939
-0.0002
NH2
O
H3CO
8a
1
2
OCH3
9
8
11
3
6
4a
4
CH3
10
OH
O
1.03
1.00
3.07
3.29
3.20
f1 (ppm)

B.

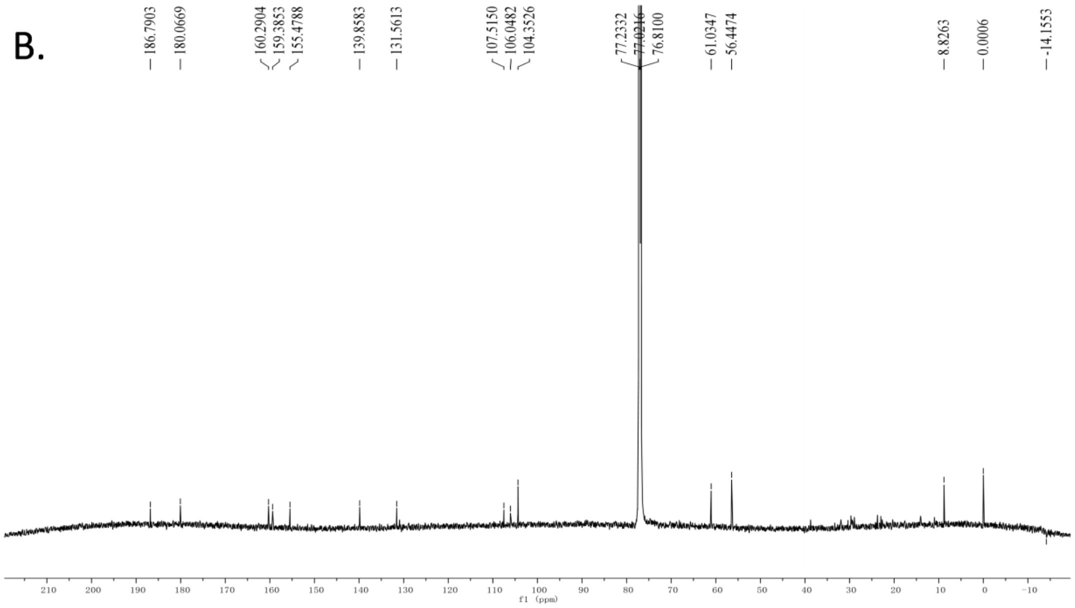
186.7903
180.0669
160.2904
159.3853
155.4788
139.8583
131.5613
107.5150
106.0482
104.3526
77.2332
77.0216
76.8100
61.0347
56.4474
8.8263
0.0006
-14.1553
f1 (ppm)

C.

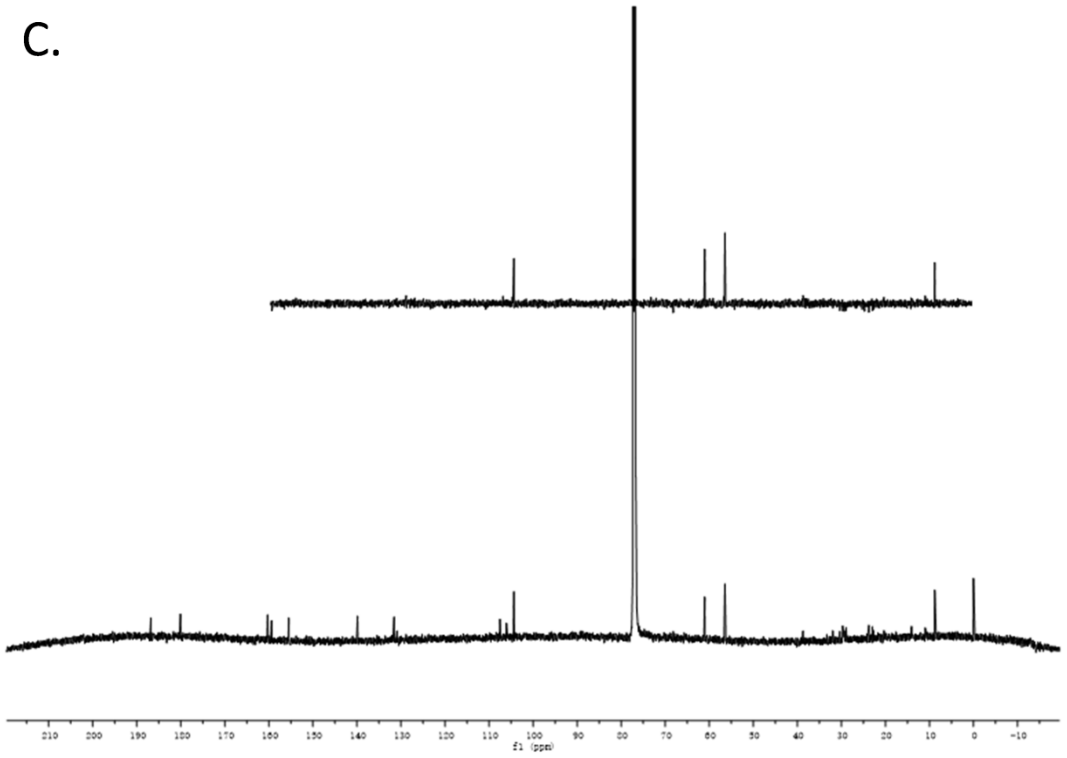

D.

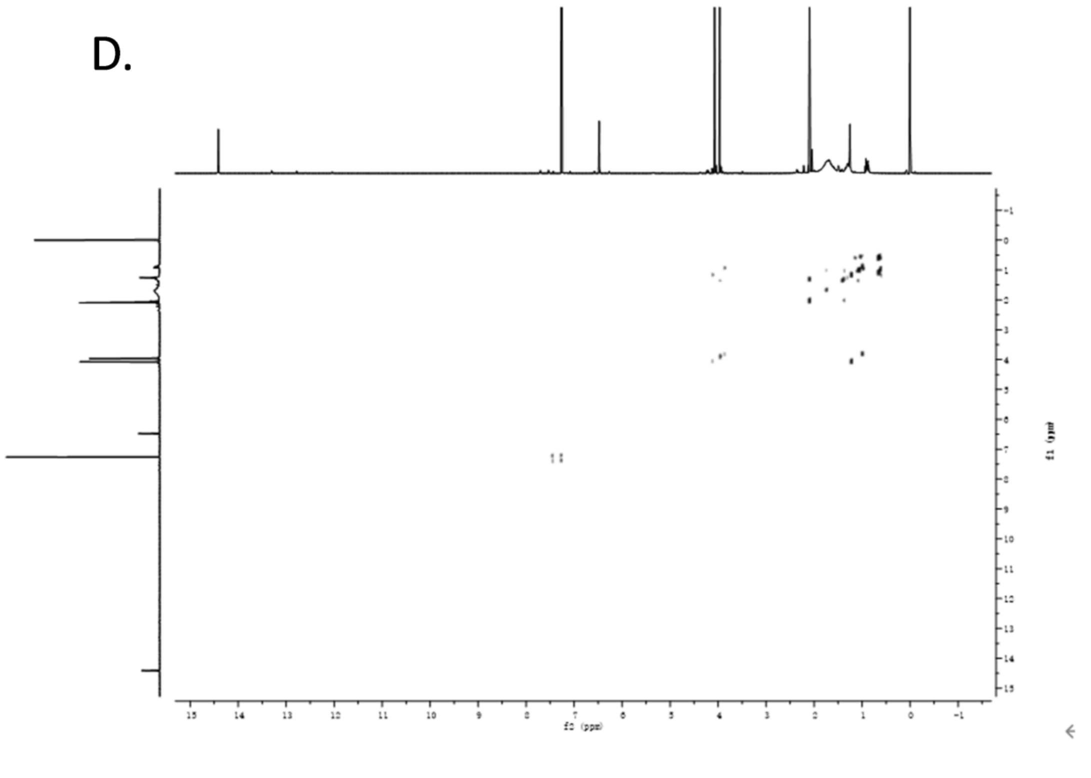

E.

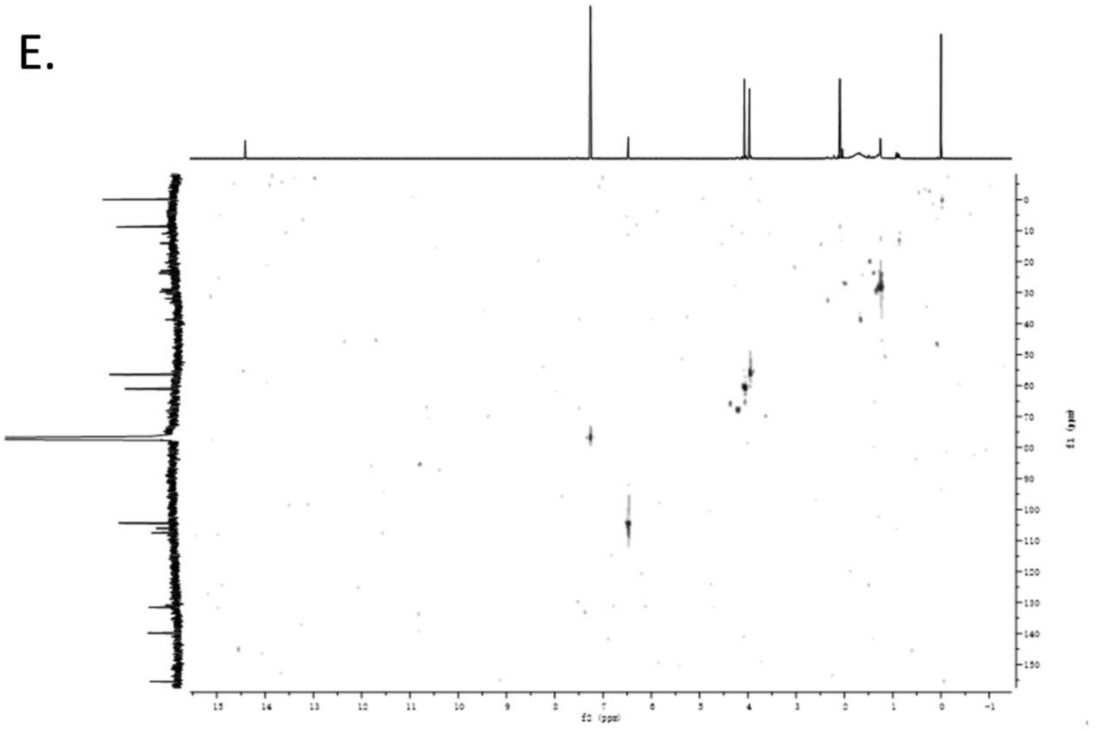

F.

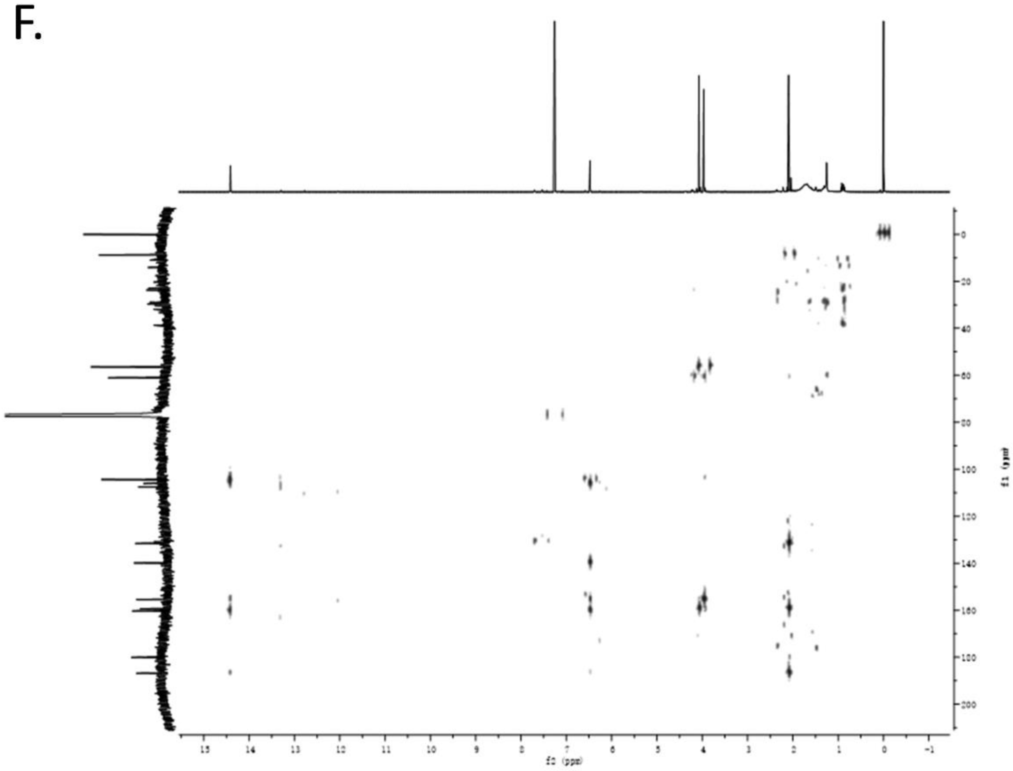

Figure S3. NMR spectra of flaviogeranin B1 (**1**). (**A**) ^{1}H NMR spectrum. (**B**) ^{13}C NMR spectrum. C. DEPT 135 spectrum. D. ^{1}H-^{1}H COSY spectrum. E. HSQC spectrum. F. HMBC spectrum.

A.

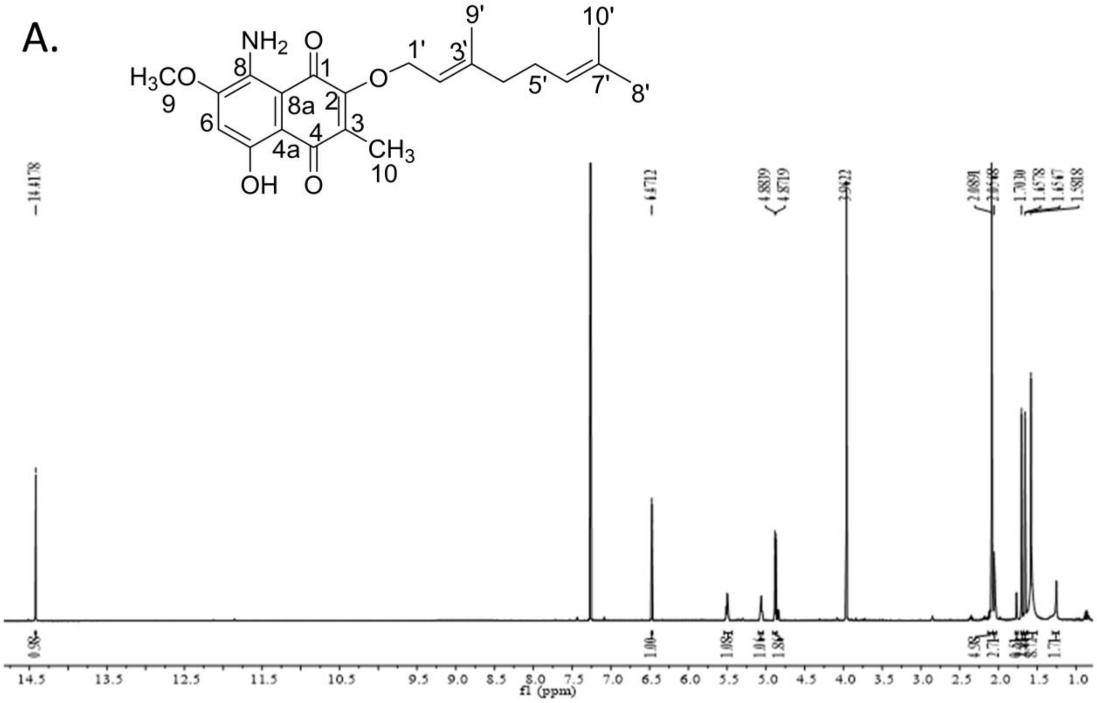

NH2
O
1
1'
3'
9'
10'
O
H3CO
8
2
8a
5'
7'
8'
9
6
4
3
CH3
4a
10
OH
O
14.4178
6.4712
4.8839
4.8719
3.9422
2.0891
1.7030
1.6578
1.6167
1.5818
0.58
1.00
1.08
1.01
1.80
4.58
2.71
1.71
14.5
13.5
12.5
11.5
10.5
9.5
9.0
8.5
8.0
7.5
7.0
6.5
6.0
5.5
5.0
4.5
4.0
3.5
3.0
2.5
2.0
1.5
1.0
f1 (ppm)

B.

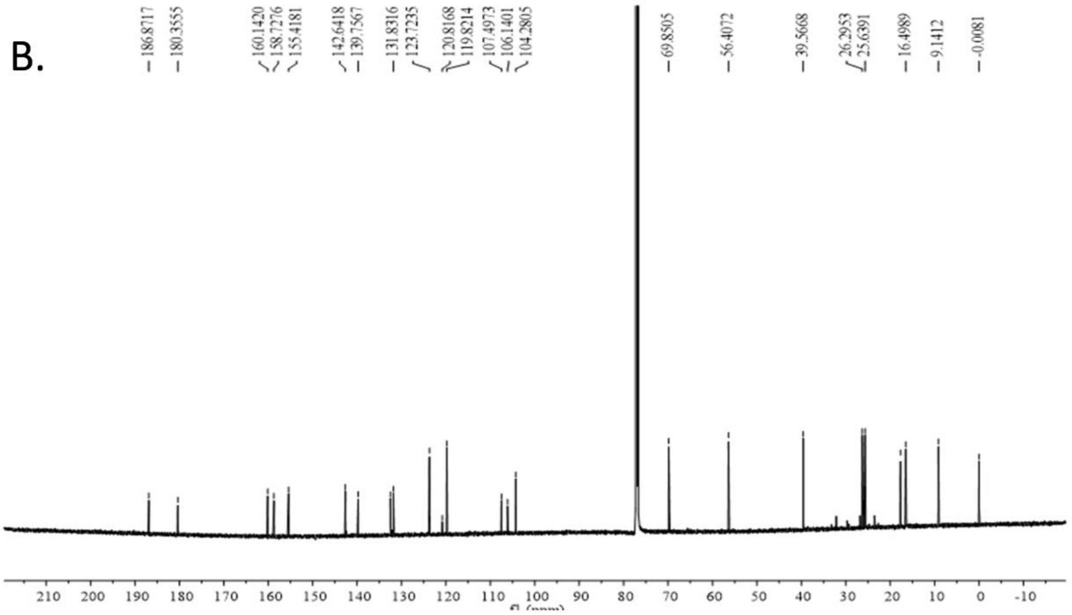

186.8717
180.3555
160.1420
158.7276
155.4181
142.6418
139.7567
131.8316
123.7235
120.8168
119.8214
107.4973
106.1401
104.2805
69.8505
56.4072
39.5668
26.2953
25.6391
16.4989
9.1412
-0.0081
210
200
190
180
170
160
150
140
130
120
110
100
90
80
70
60
50
40
30
20
10
0
-10

C.

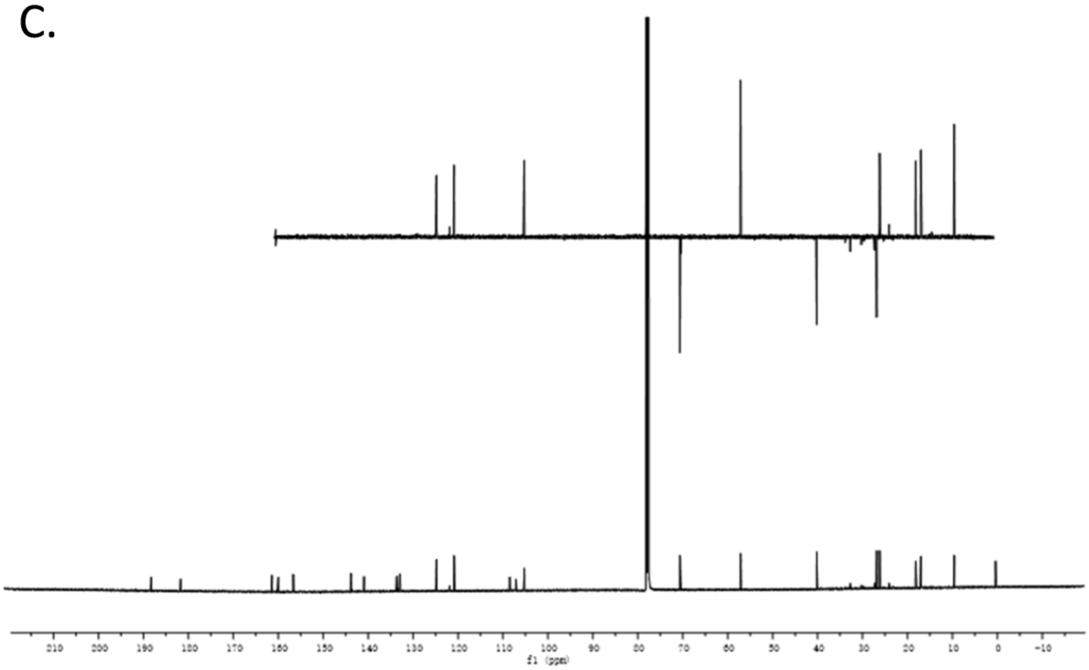

D.

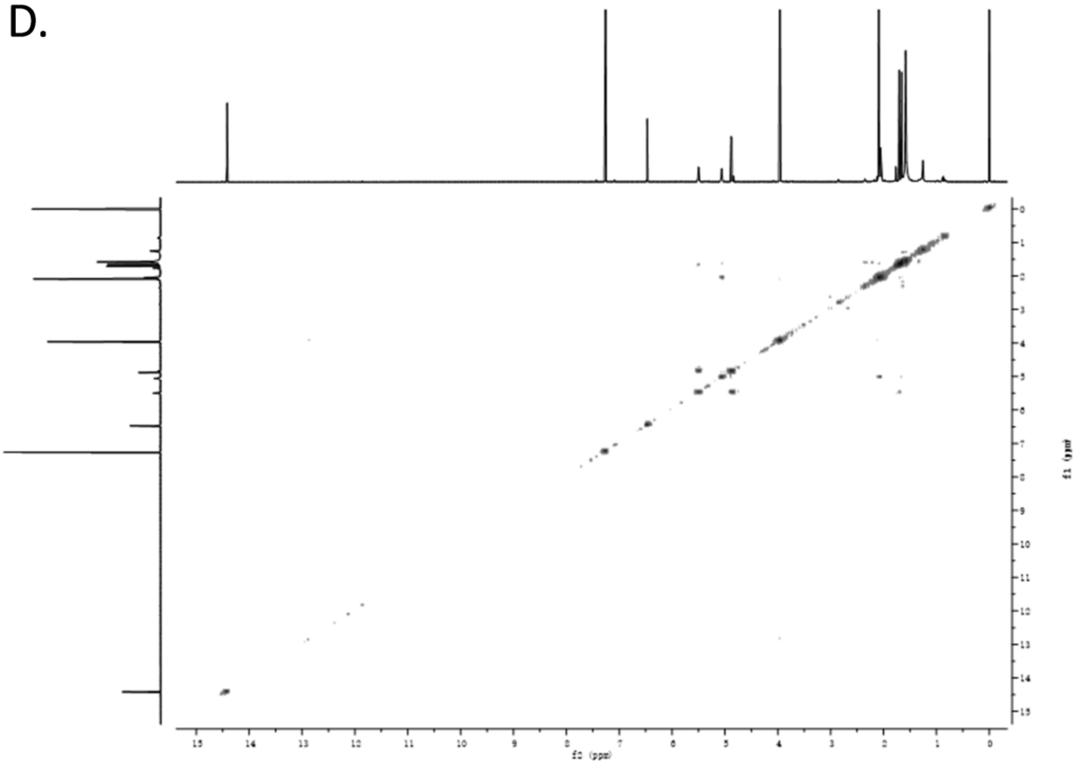

E.

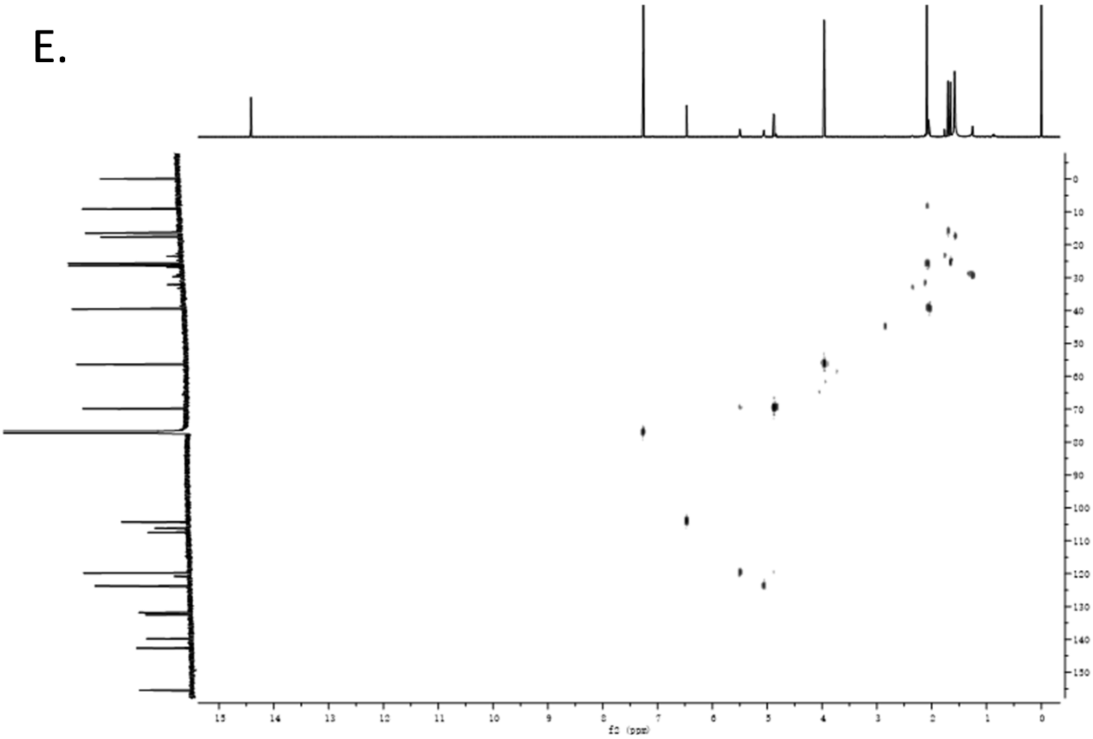

F.

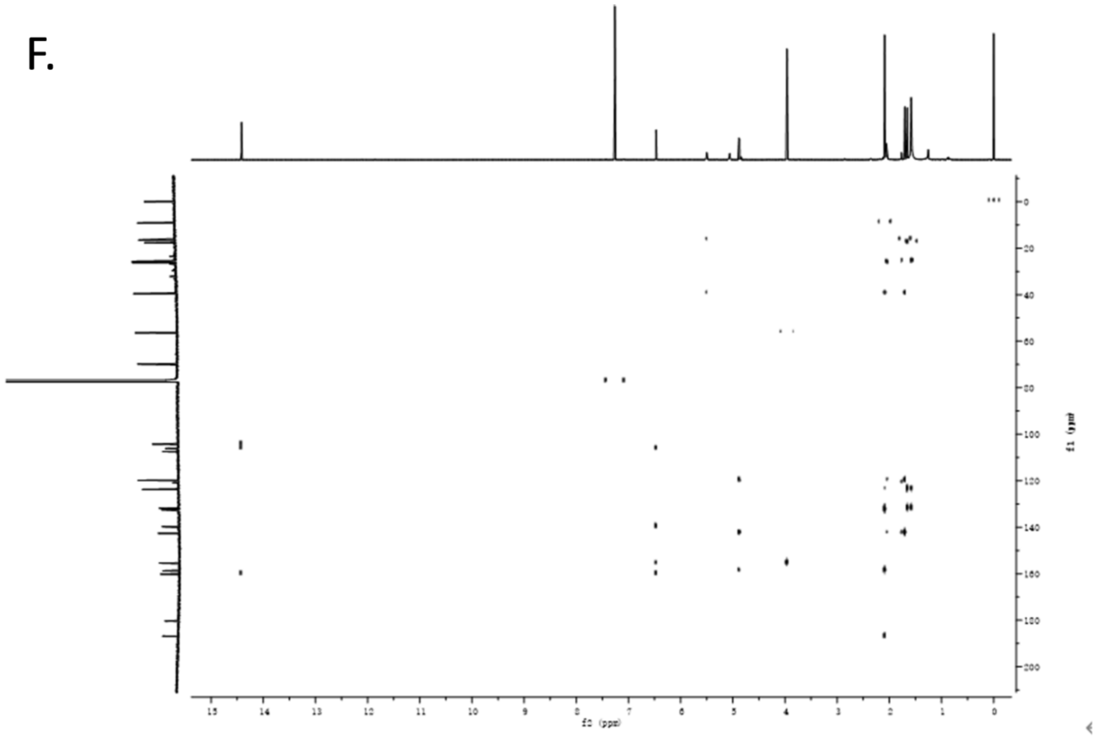

Figure S4. NMR spectra of flaviogeranin B2 (**2**).

A. ^{1}H NMR spectrum. B. ^{13}C NMR spectrum. C. DEPT 135 spectrum. D. ^{1}H-^{1}H COSY spectrum. E. HSQC spectrum. F. HMBC spectrum.

A.

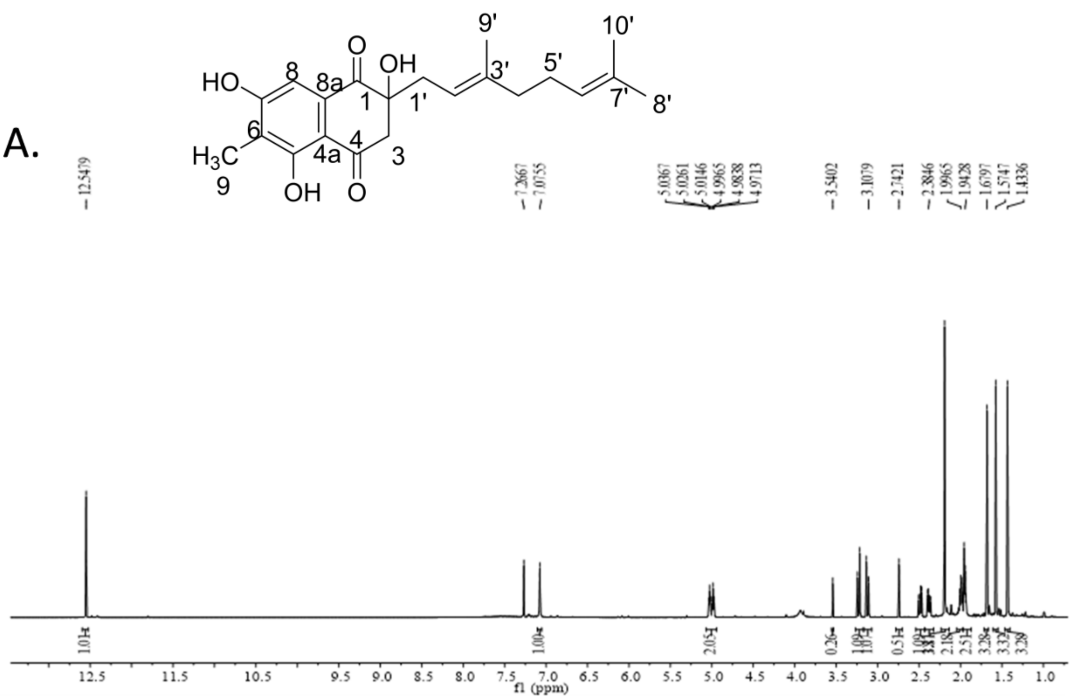

HO
OH
OH
O
O
H_3C
8
8a
1
1'
3'
5'
7'
8'
9'
10'
6
4a
4
3
9
12.5479
7.2667
7.0755
5.0367
5.0261
5.0146
4.9965
4.9838
4.9713
3.5402
3.1079
2.7421
2.3846
1.9965
1.9428
1.6797
1.5747
1.4336
1.01
1.00
2.05
0.26
1.09
1.07
0.51
2.18
2.51
3.28
3.32
3.29
12.5
11.5
10.5
9.5
9.0
8.5
8.0
7.5
7.0
6.5
6.0
5.5
5.0
4.5
4.0
3.5
3.0
2.5
2.0
1.5
1.0
f1 (ppm)

B.

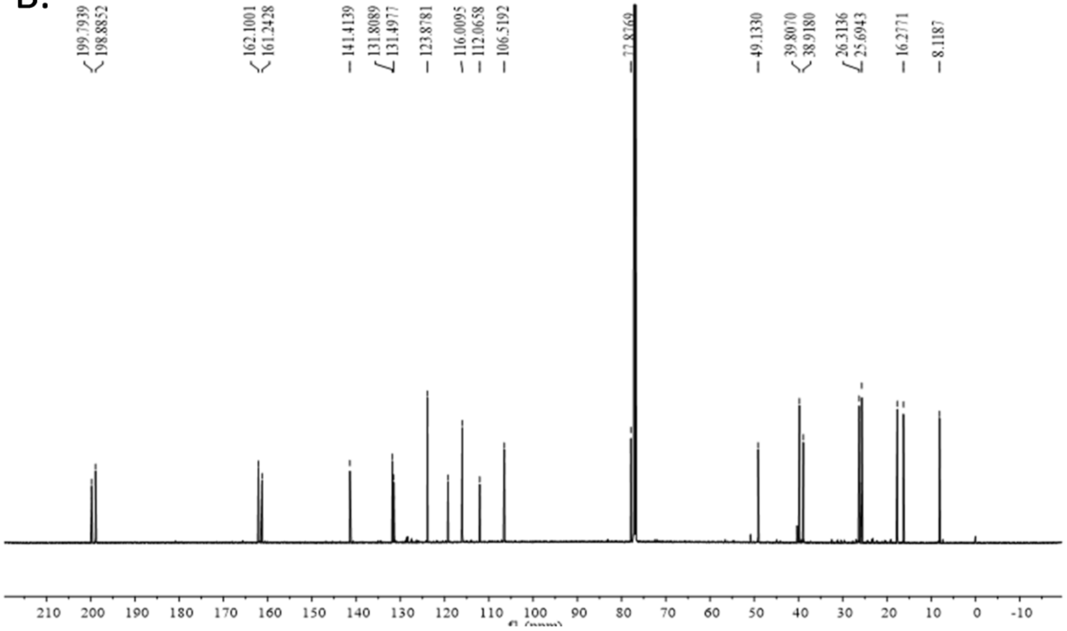

199.7939
198.8852
162.1001
161.2428
141.4139
131.8089
131.4977
123.8781
116.0095
112.0658
106.5192
49.1330
39.8070
38.9180
26.3136
25.6943
16.2771
8.1187
210
200
190
180
170
160
150
140
130
120
110
100
90
80
70
60
50
40
30
20
10
0
-10

C.

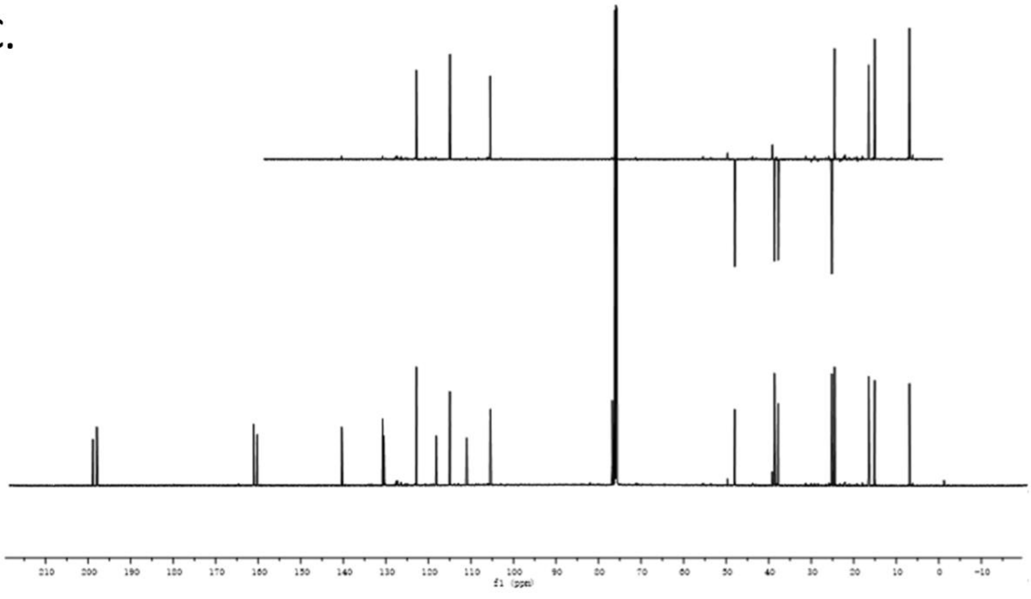

D.

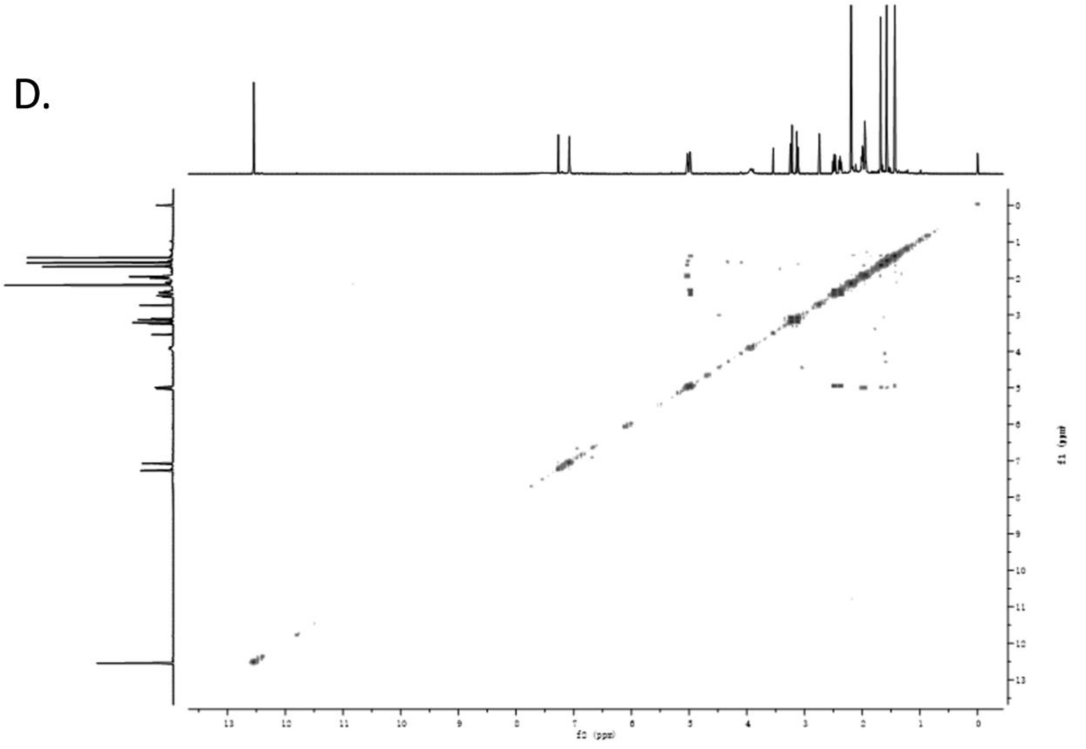

E.

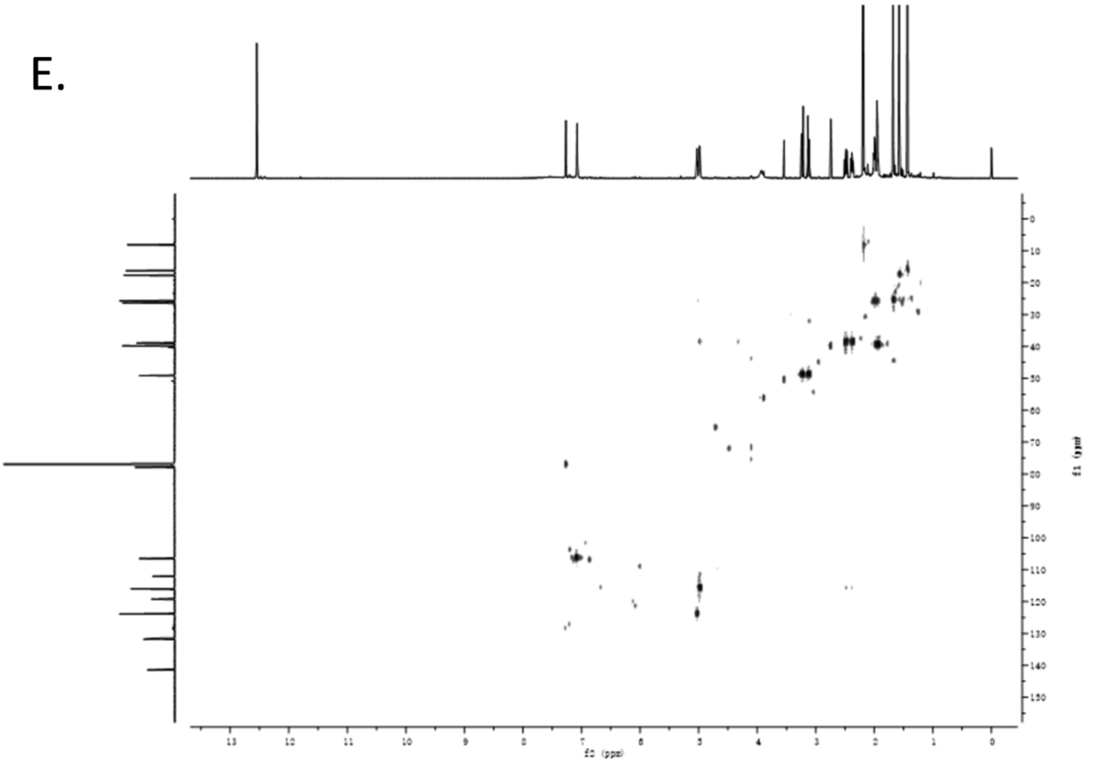

F.

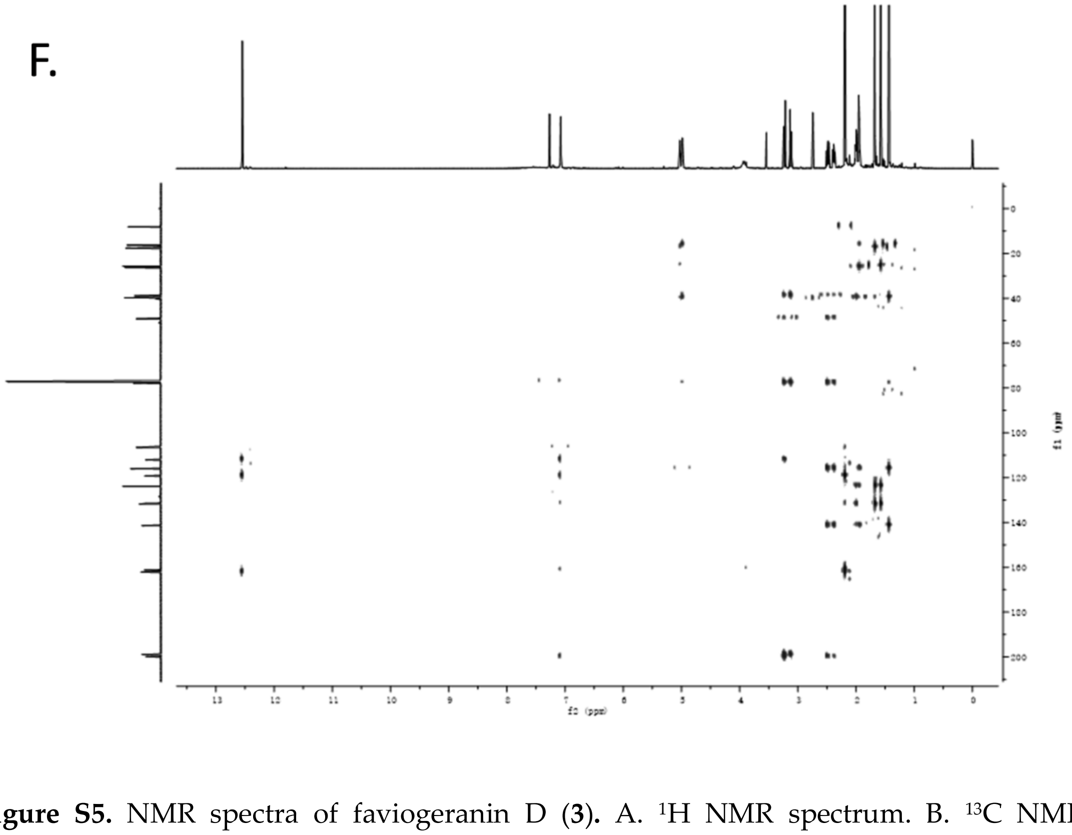

Figure S5. NMR spectra of faviogeranin D (**3**)**.** A. ^{1}H NMR spectrum. B. ^{13}C NMR spectrum. C. DEPT 135 spectrum. D. ^{1}H-^{1}H COSY spectrum. E. HSQC spectrum. F. HMBC spectrum.

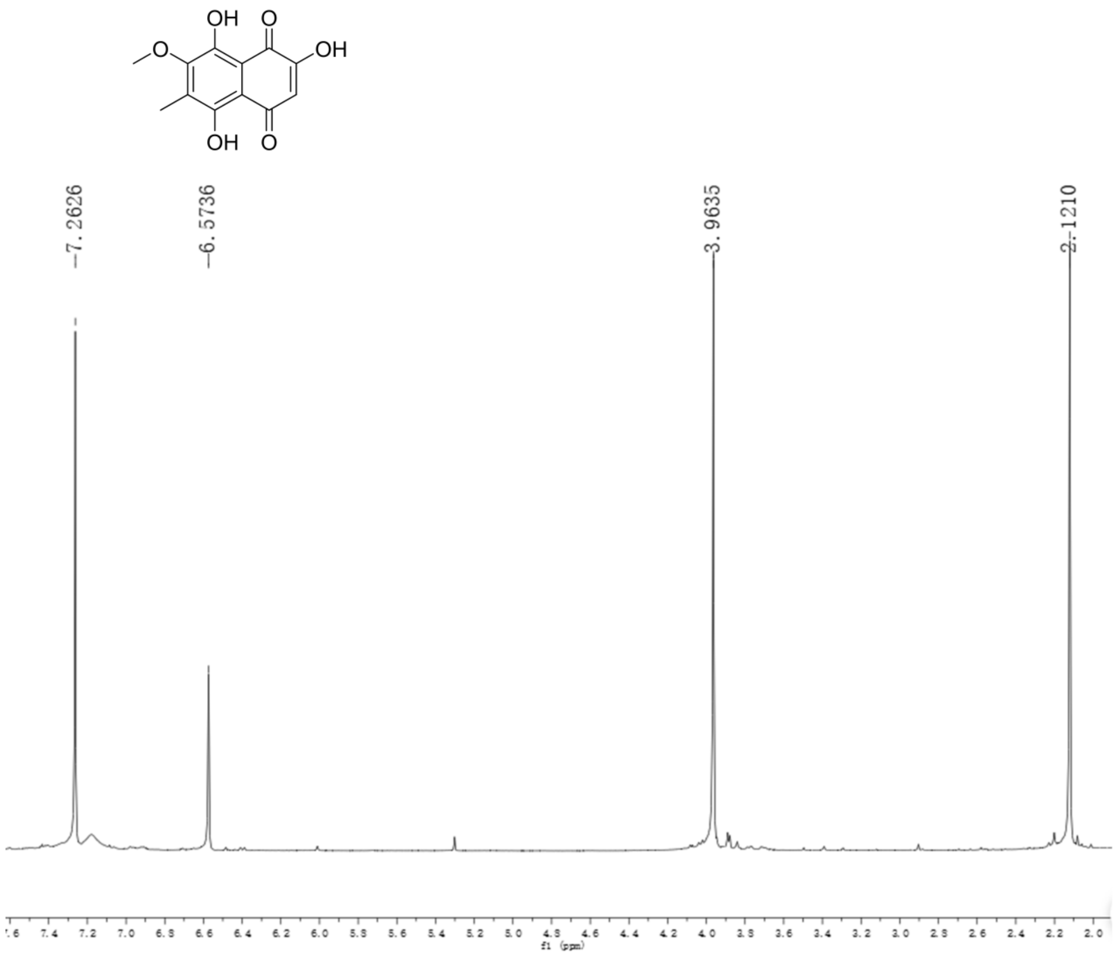

Figure S6. ^{1}H NMR spectrum of flaviogeranin C1 (**4**).

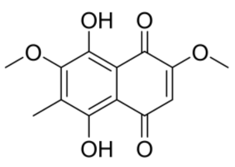

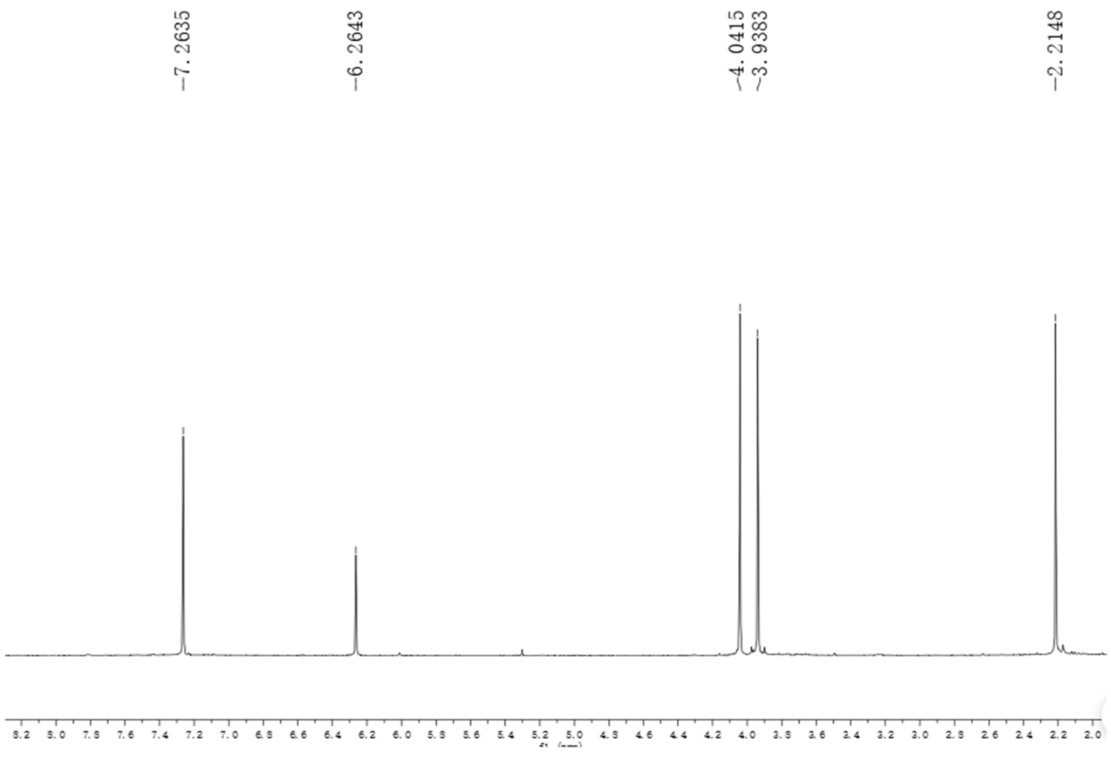

Figure S7. ^{1}H NMR spectrum of flaviogeranin C2 (**5**).

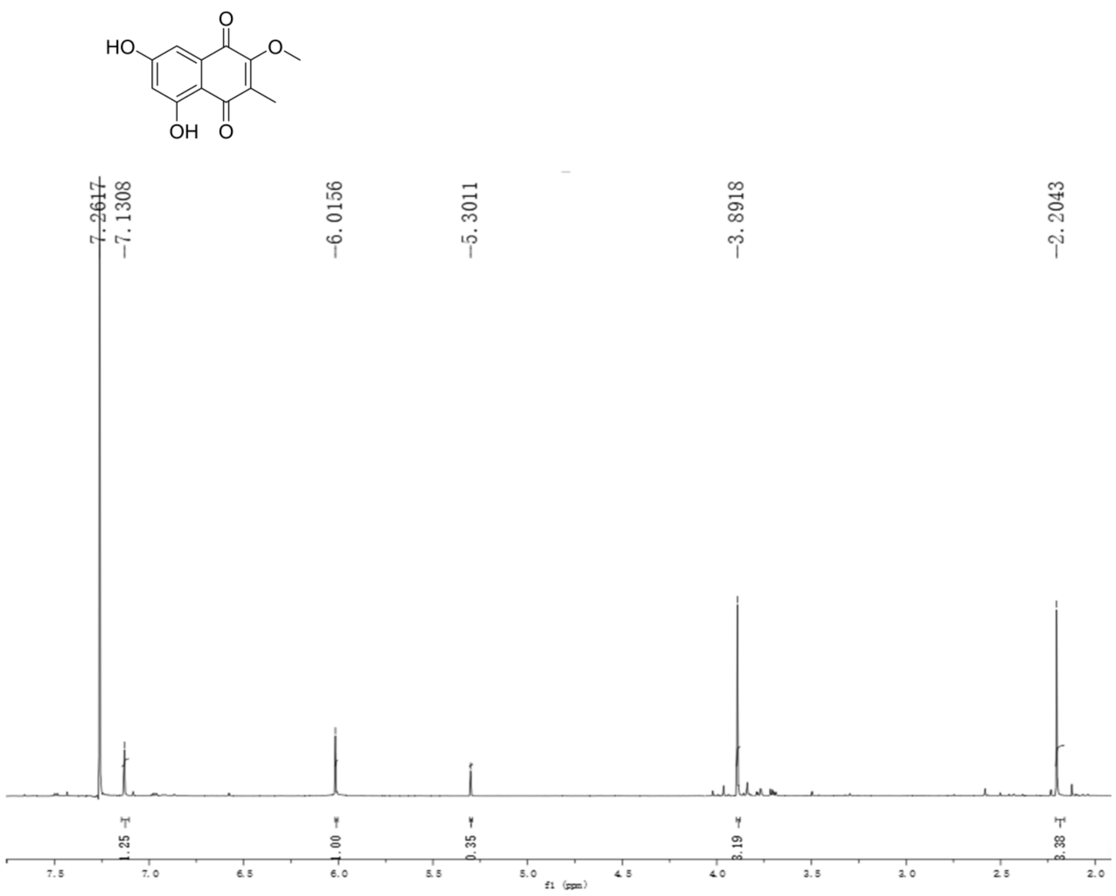

Figure S8. ${}^{1}H$ NMR spectrum of flaviogeranin A1 (**6**).

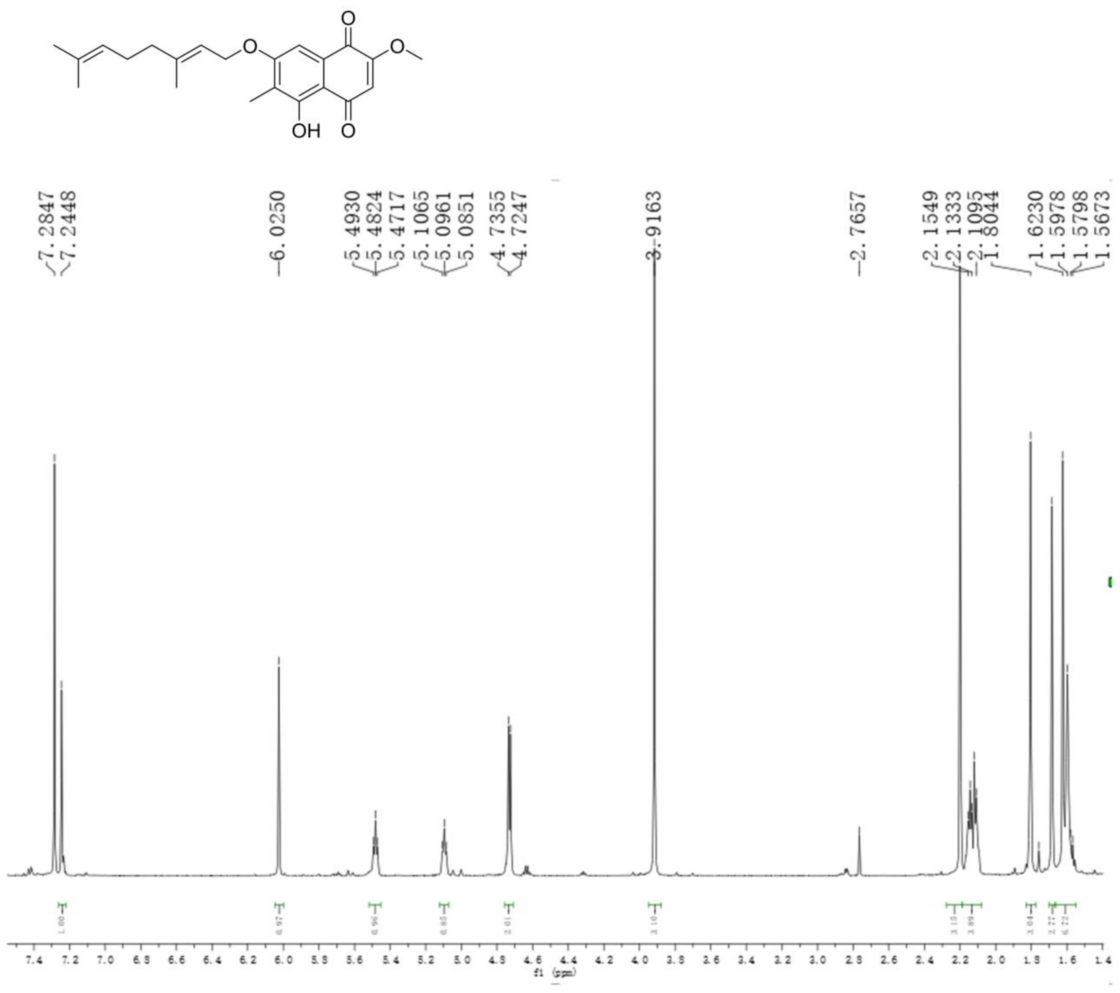

Figure S9. ^{1}H NMR spectrum of flaviogeranin A (**7**).

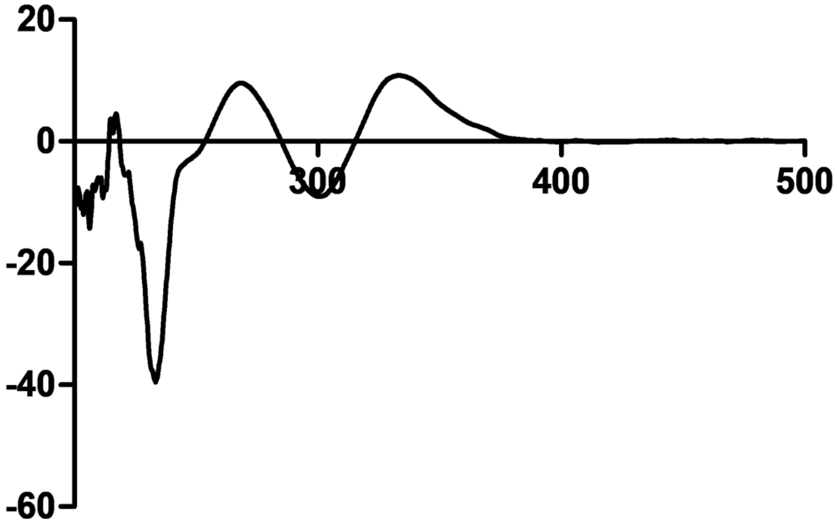

Figure S10. CD spectrum of flaviogeranin D (**3**).

Table S1. HR-ESI MS of known compounds **4**-**7**.

**Compounds**	**Expected** m/z **[M + H]**$^{+}$	**Found** m/z **[M + H]**$^{+}$
**4**	251.0550	251.0451
**5**	265.0707	265.0681
**6**	235.0601	235.0602
**7**	371.1853	371.1858
